# Establishment of reference ranges for disturbance coefficient (DC) and edema coefficient (EC) in neonates: a non-invasive cerebral edema monitoring study

**DOI:** 10.3389/fneur.2026.1712487

**Published:** 2026-10-22

**Authors:** Bing Wu, Jingjing Jia, Dongying Qu, Jun Ma, Yanqiu Xia, Mo Li, Juncheng Zhu, Ming Yang, Bing Wei

**Affiliations:** 1 Department of Neonatology, Northern Theater Command General Hospital, Shenyang, Liaoning, China; 2 Post-graduate College, Jinzhou Medical University, Jinzhou, China; 3 Department of Obstetrics and Gynecology, Northern Theater Command General Hospital, Shenyang, Liaoning, China

**Keywords:** disturbance coefficient (DC), edema coefficient (EC), neonates, reference ranges, the non-invasive cerebral edema dynamic monitor

## Abstract

**Objective:**

To establish reference ranges for the disturbance coefficient (DC) and edema coefficient (EC) in neonates without traumatic brain injury, based on head circumference, weight, and body length, to provide early warnings for neonatal brain injury.

**Methods:**

Neonates were collected from the rooming-in unit of Northern Theater Command General Hospital, China, between June and December 2024. The non-invasive cerebral edema dynamic monitor was used to measure DC and EC at 24, 48, and 72 h after birth. Correlations between DC, EC and clinical parameters (head circumference, weight, body length, sex, gestational age, and mode of delivery) were analyzed statistical methods, including Kruskal–Wallis test, Spearman's correlation test, and Mann–Whitney test.

**Results:**

Totally 150 neonates with a gestational age of over 36 weeks were recruited, the median gestational age was 38.4 weeks, with 73 male (48.7%). And the predominant mode of delivery was cesarean section (82.7%). The median (interquartile range, IQR) DC increased significantly from 33.40 (28.45–41.60) at 0–1 day to 39.50 (32.58–46.20) at 1–2 days and 41.65 (35.30–46.90) at 2–3 days after birth, while EC decreased from 16.20 (9.88–20.38) to 10.95 (5.58–16.90) and 9.45 (5.20–14.73), respectively. DC was positively correlated with head circumference (*r* = 0.485, *P* < 0.001), weight (*r* = 0.499, *P* < 0.001), and body length (*r* = 0.427, *P* < 0.001), while EC was negatively correlated with these parameters (head circumference: *r* = −0.480, *P* < 0.001; weight: *r* = −0.492, *P* < 0.001; body length: *r* = −0.423, *P* < 0.001). No significant correlations were found between DC, EC and sex, gestational age, or mode of delivery.

**Conclusions:**

In neonates over 36 weeks, DC significantly increased from 0–1 day to 1–2 and 2–3 days after birth, while EC decreased. DC showed a positive correlation with head circumference, weight, and body length, while EC exhibited a negative correlation. DC and EC were not affected by sex, mode of delivery, and gestational age.

**Clinical Trial Registration:**

identifier: ChiCTR2500095381.

## Introduction

The primary cause of neonatal brain injury is perinatal hypoxia-ischemia. Since neonates have immature cerebral autoregulatory function, particularly preterm infants, their cerebral blood vessels cannot maintain stable blood flow when hypoxia-ischemia occurs, which leads to the formation of “pressure-passive cerebral blood flow” and thus induces brain injury.

Term infants mostly present with hypoxic-ischemic encephalopathy (HIE), whereas preterm infants often develop complications such as periventricular-intraventricular hemorrhage (PVH-IVH) and periventricular leukomalacia (PVL), and are accompanied by concurrent cerebral edema in these conditions (1–3). Most IVH occurs within the first 72 h after birth (early IVH) and progresses rapidly within the first week of life (1). This signifies the importance of early identification of these neonatal brain injuries to enable the establishment of targeted care bundles in the reduction of neonatal brain injury.

The non-invasive cerebral edema dynamic monitor is a instrument that utilizes electrical bioimpedance technology and electromagnetic principles to monitor dynamic changes in intracranial tissue (4). When changes occur in brain tissue, its main output parameters, edema coefficient (EC) and disturbance coefficient (DC), will also change accordingly, reflecting the intracranial conditions and providing timely warnings (5, 6). The disturbance coefficient (DC) mainly consisted of mathematical compositions of waveform amplitude and phase, and it reflected real-time and dynamic alterations in the electromagnetic properties and structural status of brain tissues. When intracranial hemorrhage or edema occurred in brain tissues, the dielectric constant of local tissues changed, which affected the propagation velocity of electromagnetic waves and further caused the vertical shift of waveform phases to indicate variations in intracranial tissues. The edema coefficient (EC) mainly represented the accumulation level of extracellular fluid in brain cells and displayed the severity of tissue edema through electrical signals. Compared with other tools for monitoring craniocerebral injury, such as computed tomography, magnetic resonance imaging, and transcranial Dopple, they not only reduce concerns about radiation exposure and high costs but also provide real-time and dynamic bedside monitoring (7–9). And invasive monitoring methods (i.e., lumbar puncture manometry and intraventricular tube manometry) have potential risks of intracranial infection, which limits their clinical applications (10–12). Therefore, non-invasive cerebral edema monitor, as a convenient, continuous, and non-invasive brain monitoring instrument, is increasingly being applied in clinical practice (13–16).

In recent years, non-invasive cerebral edema monitor has demonstrated significant advancements in the management of adult cerebral infarction, cerebral hemorrhage, and traumatic brain injury (17–19). This technology is being utilized for pediatric traumatic brain injury, encompassing the assessment of brain edema and intracranial pressure, as well as the evaluation of injury severity and prognosis (20, 21). Previous studies had shown that the DC of non-invasive cerebral edema monitor varied among children of different ages (22, 23). However there are currently no reference ranges for DC and EC in neonates without traumatic brain injury. Brain damage to the immature brains of neonates has profound and lasting effects, impacting both immediate and future neurological functions. To enhance early identification and prognosis of neonatal brain injury, it is essential to clearly define the ranges of the DC and EC in neonates (17, 24, 25). This study is the first to establish reference ranges for the DC and EC of neonates without traumatic brain injury based on head circumference, weight, and body length, in order to provide early warning for the occurrence of neonatal brain injury and obtain timely treatment.

## Materials and methods

### Study design

This study recruited 150 neonates from the rooming-in unit of Northern Theater Command General Hospital in China, between June 2024, and December 2024. Inclusion criteria include: (1) age between 0 and 28 days, sex not limited; (2) gestational age >36 weeks. Exclusion criteria include: (1) scalp hematoma, skull defect, head skin damage, and rash; (2) allergy to electrode stickers; (3) Infants may have conditions affecting cerebral blood flow such as hypoxia, asphyxia, respiratory distress or acidosis, as well as congenital neurodevelopmental abnormalities; (4) unable to cooperate. And we recorded general information of all participants, such as sex, gestational age, age, weight, and body length.

Ethical approval was provided by the human research ethics committees of the Northern Theater Command General Hospital [No.Y(2024)348)] and registered in the Chinese Clinical Trial Registry (ChiCTR2500095381). All parents gave written informed consent.

### Data acquisition and acquisition

The data was obtained using non-invasive cerebral edema dynamic monitor (BORN-BE-IIIA, ChongQing). (1) The skin was exposed and disinfect superior to the bilateral earlobes of the newborn, apply conductive gel to the exposed skin, and attach four specialized electrode stickers; (2) Before monitoring, check the integrity and expiration date of the electrode stickers, connect the electrode stickers to the data cable, and enter subject's information (age, sex, body length, weight, head circumference). Each subject is measured for 15 min each time. The monitor automatically generated one disturbance coefficient (DC) value and one edema coefficient (EC) value at intervals of approximately 1 to 2 min. Roughly 10 DC values and 10 EC values were obtained throughout the 15-min monitoring period. The mean values of DC and EC from each measurement were adopted as the final values for each subject; (3) Monitor once at 24 h, 48 h, and 72 h after birth, using the same method (Figure 1). Take the mean DC and EC values of each time as the final DC and EC values of the subjects.

**Figure 1:**
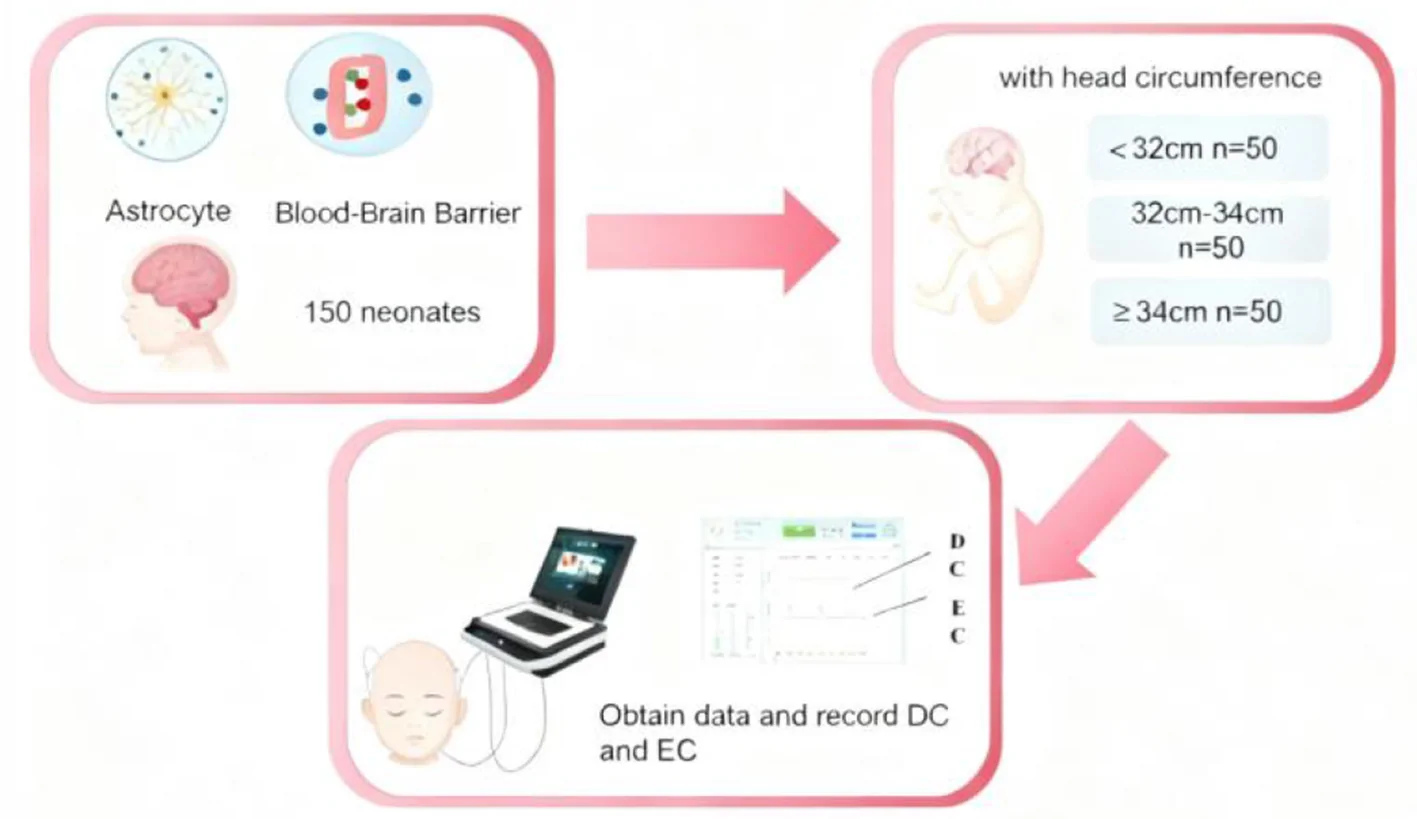
Flow chart of the study neonates and design (Created with BioRender.com).

### Sample size estimation

Based on preliminary experimental results on 45 neonates without traumatic brain injury, we found that the EC and DC were correlated in neonates with head circumference (< 32 cm, 32–34 cm, ≥34 cm). In this trial, the test power was taken as β = 0.1, α = 0.05, the sample size of the three groups was equal, and was tested on both sides. Using PASS 23.0, we calculated *n* = 11. Therefore, in this study, we took *n* = 50 per group to minimize sampling error and ensure stable reference interval estimation, with a total sample size of 150.

### Statistical analysis

Data from 150 neonates were analyzed using descriptive statistics, Generalized Linear Mixed Model, and correlation analysis. Continuous variables were summarized by medians and IQR, while categorical variables were described by frequencies and percentages. Differences in DC and EC across time points (0–1 day, 1–2 days, 2–3 days) and pairwise comparisons were assessed using the Generalized Linear Mixed Model (GLMM) with an autocorrelated covariance structure. In the pilot experiment, we hypothesized a normal distribution of the results; however, the final data exhibited a skewed distribution, so we adjusted the statistical method and adopted the Mann-Whitney test for pairwise comparisons. Spearman's correlation was used to evaluate associations between DC, EC and head circumference, weight, and body length. *P*-values < 0.05 were considered significant. Analyses were performed using SPSS 27.0 and R software.

## Results

### Subject characteristics

This study included 150 subjects, [median (IQR) gestational age, 38.4 (37.6–39.1) weeks; 73 male (48.7%)]. The medians (IQR) head circumference and weight were 33 (31–34) cm and 3,050 (2,560–3,410) g respectively. Cesarean delivery was the predominant mode of delivery [124 of 150 (82.7%)]. Characteristics of the subject (Table 1).

**Table 1:** Subject characteristics The DC and EC in different ages.

Variable	All No./total No.	Median (IQR) or %
Sex, male	73/150	48.7%
Cesarean delivery	124/150	82.7%
Gestational age, week	150/150	38.4 (37.6–39.1)
Head circumference, cm	150/150	33 (31–34)
Weight, g	150/150	3,050 (2,560–3,410)
Body length, cm	150/150	52 (51–54)
Within 0–1 day after birth		
DC	150/150	33.40 (28.45–41.60)
EC	150/150	16.20 (9.88–20.38)
Within 1–2 days after birth		
DC	150/150	39.50 (32.58–46.20)
EC	150/150	10.95 (5.58–16.90)
Within 2–3 days after birth		
DC	150/150	41.65 (35.30–46.90)
EC	150/150	9.45 (5.20–14.73)

DC increased significantly while EC decreased significantly at 0–1 days, 1–2 days and 2–3 days, and there were statistically significant differences in DC and EC across these three time points.

### The DC and EC in different age

The DC and EC of neonates with different age showed significant differences between groups (Figure 2). Figure 2 showed that the DC and EC were correlated with age. The DC and EC of neonates were significantly different between aged 0–1 day group and aged 1–2 days group (*P* < 0.0001) and aged 1–2 days group and aged 2–3 days group and aged 0–1 day group and aged 2–3 days group. The DC increased extremely significantly with the increase in age groups (β = 3.7457, SE = 0.4849, *t* = 7.725, *P* < 0.001), the EC decreased extremely significantly with the increase in age groups (β = −2.4500, SE = 0.3207, *t* = −7.639, *P* < 0.001).

**Figure 2:**
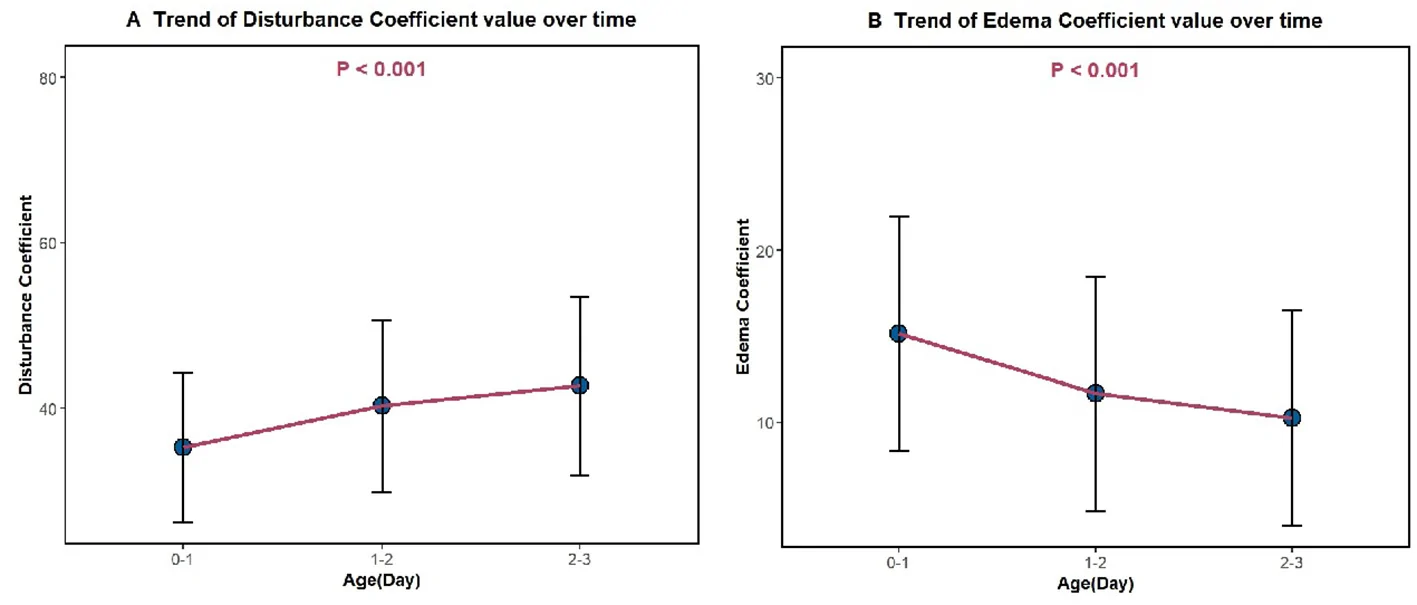
Differences in DC **(A)** and EC **(B)** among different ages. Statistical significance was assessed through the Generalized Linear Mixed Model (GLMM) with an autocorrelated covariance structure. DC, disturbance coefficient; EC, edema coefficient. *P* < 0.0001.

### The DC and EC in different head circumferences

The DC and EC of neonates with different head circumferences showed differences between groups (Figures 3, 4). Figure 3 showed that the DC and EC were correlated with head circumferences. The DC and EC of neonates were different between every two groups.

**Figure 3:**
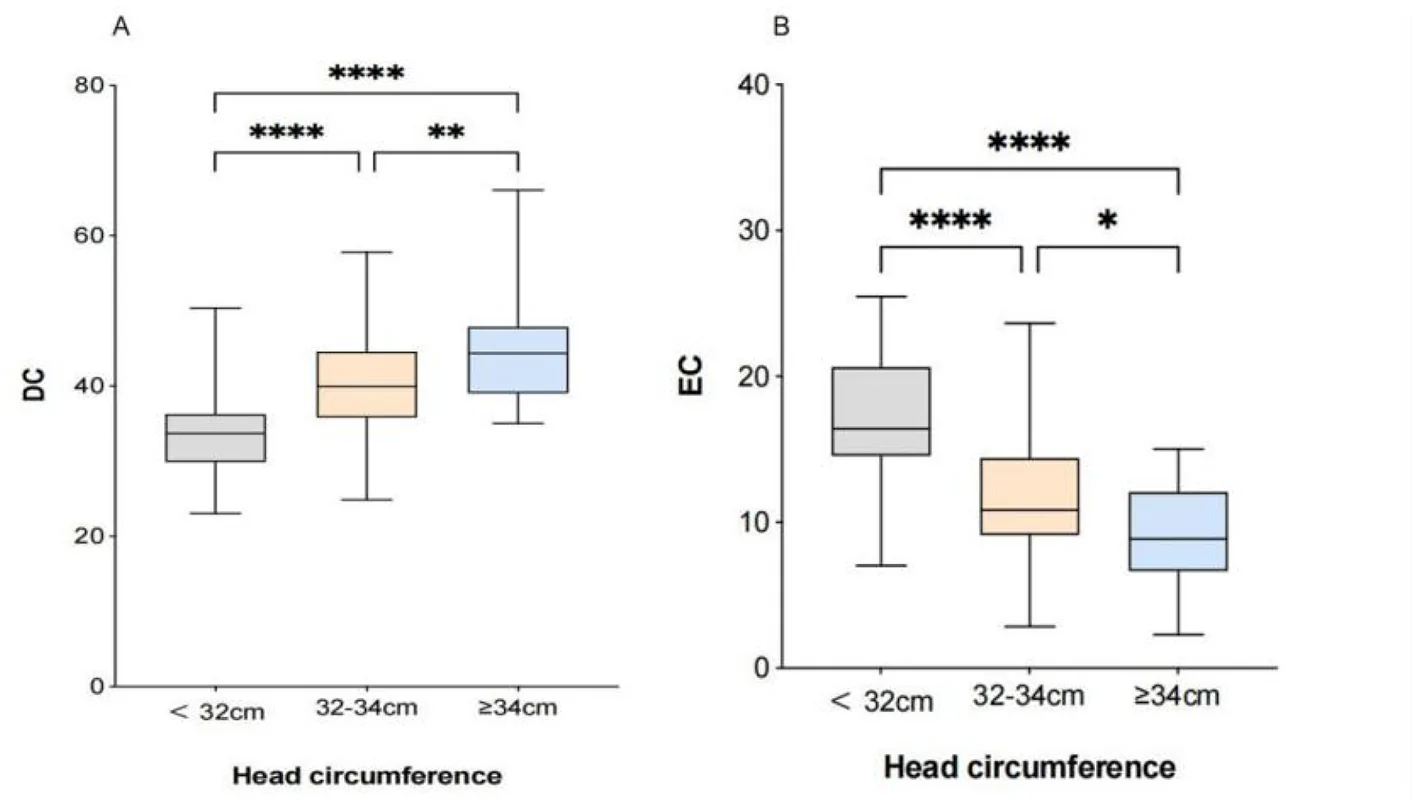
Differences in DC **(A)** and EC **(B)** among different head circumferences. Statistical significance was assessed through Kruskal–Wallis test. DC, disturbance coefficient; EC, edema coefficient. **means *P* < 0.01 after Bonferroni correction, ****means *P* < 0.0001 after Bonferroni correction.

**Figure 4:**
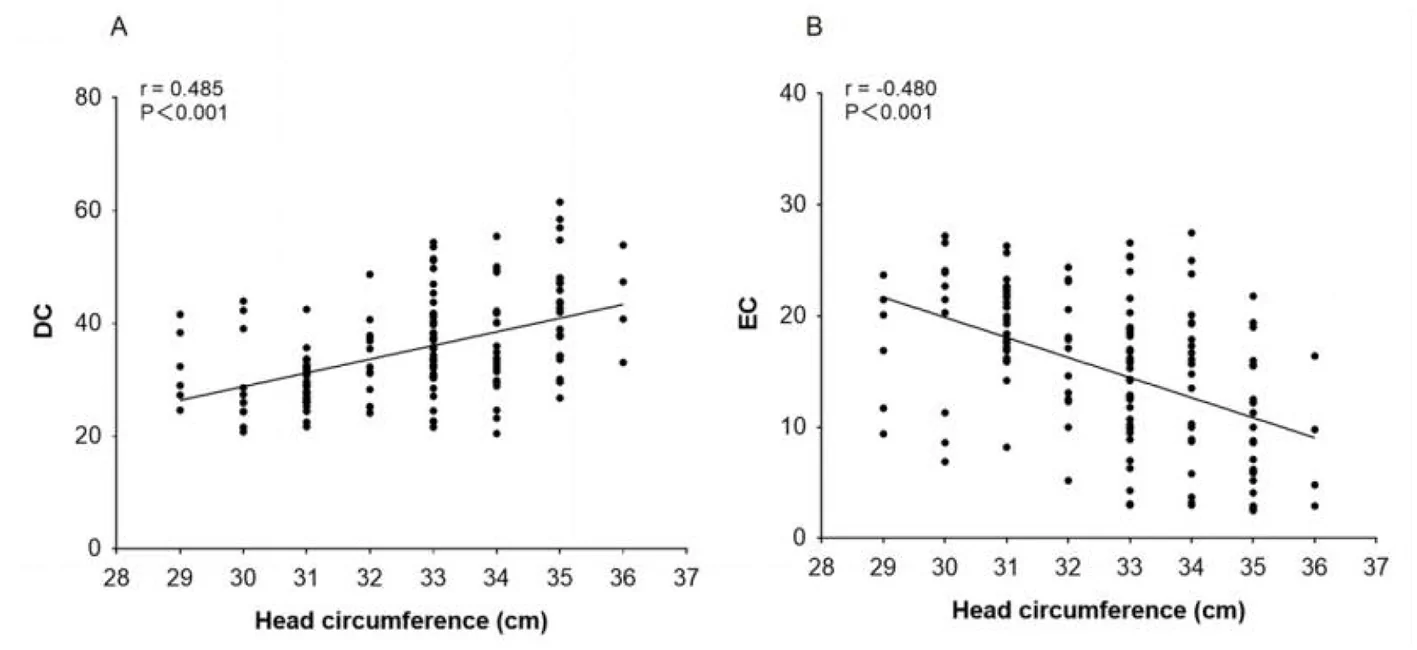
Association between head circumference and DC **(A)** and EC **(B)**. Statistical significance was assessed through Spearman's test. DC, disturbance coefficient; EC, edema coefficient.

We showed in Figure 4 that the DC and EC correlated with head circumference. As head circumference increased, the DC exhibited a notable increase (*r* = 0.485, *P* < 0.001), whereas the EC showed a corresponding decrease (*r* = −0.480, *P* < 0.001 after Bonferroni correction).

### The DC and EC in different weights

The values of DC and EC in neonates of different weights showed differences. Correlation analysis suggested a positive correlation between DC and weight (*r* = 0.499, *P* < 0.001 after Bonferroni correction). There were negatively correlated with EC in different weight groups (*r* = −0.492, *P* < 0.001 after Bonferroni correction) (Figures 5, 6).

**Figure 5:**
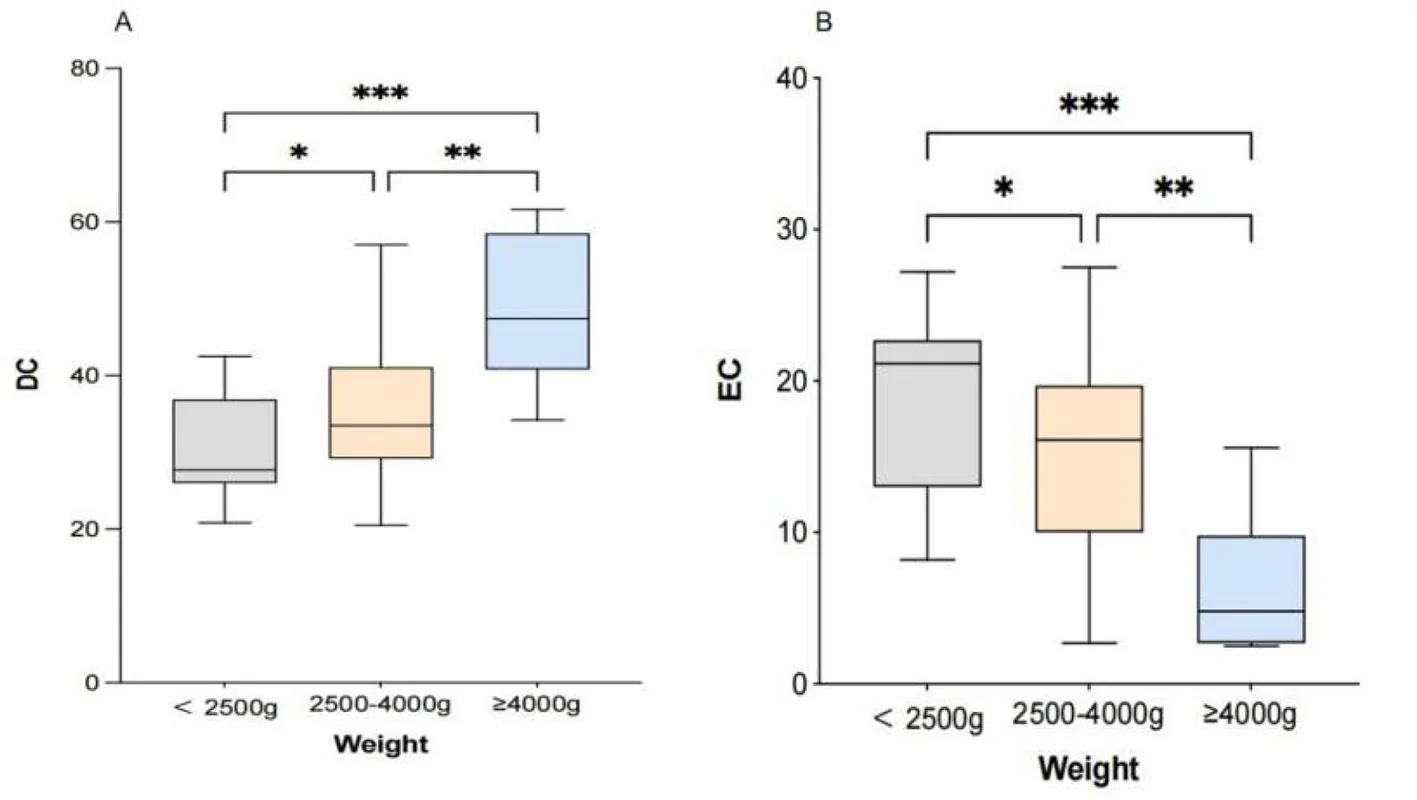
Differences in DC **(A)** and EC **(B)** among different weights. Statistical significance was assessed through Kruskal–Wallis test. DC, disturbance coefficient; EC, edema coefficient. *means *P* < 0.05, **means *P* < 0.01 after Bonferroni correction, ***means *P* < 0.001 after Bonferroni correction.

**Figure 6:**
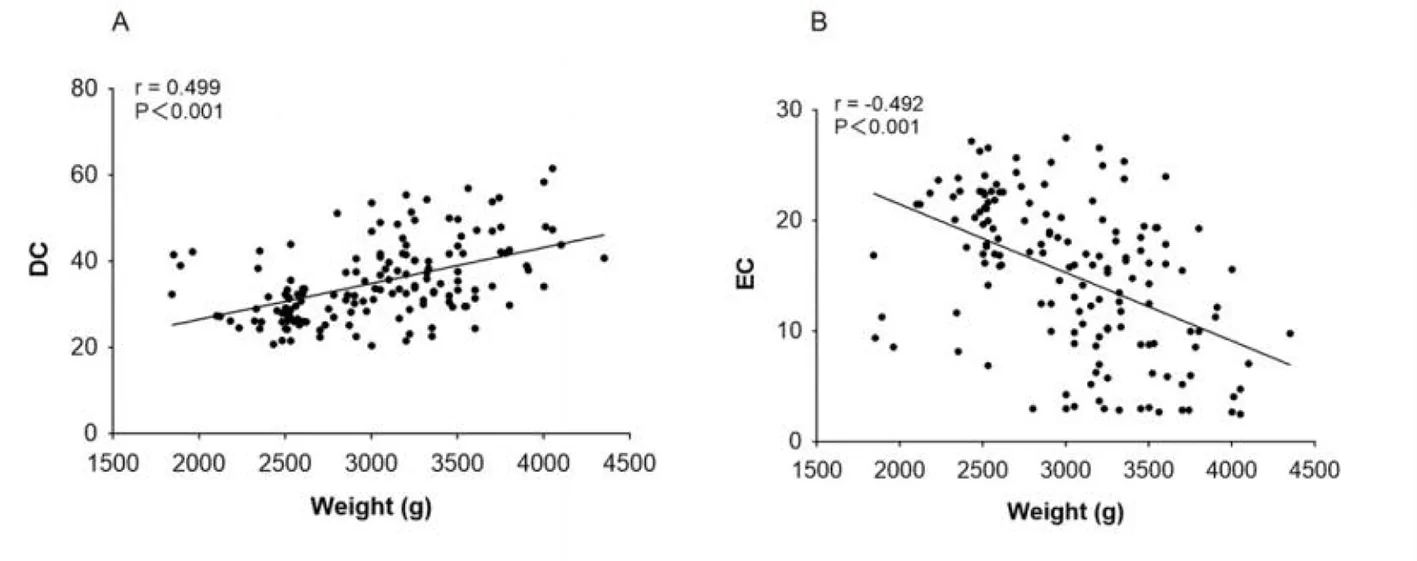
Association between weight and DC **(A)** and EC **(B)**. Statistical significance was assessed through Spearman's test. DC, disturbance coefficient; EC, edema coefficient.

### The DC and EC in different body lengths

There were differences in DC and EC among neonates of different body lengths (Figures 7, 8). DC tended to upward with increasing body length (*r* = 0.427, *P* < 0.001 after Bonferroni correction), while EC tended to downward (*r* = −0.423, *P* < 0.001 after Bonferroni correction) (Figures 7, 8).

**Figure 7:**
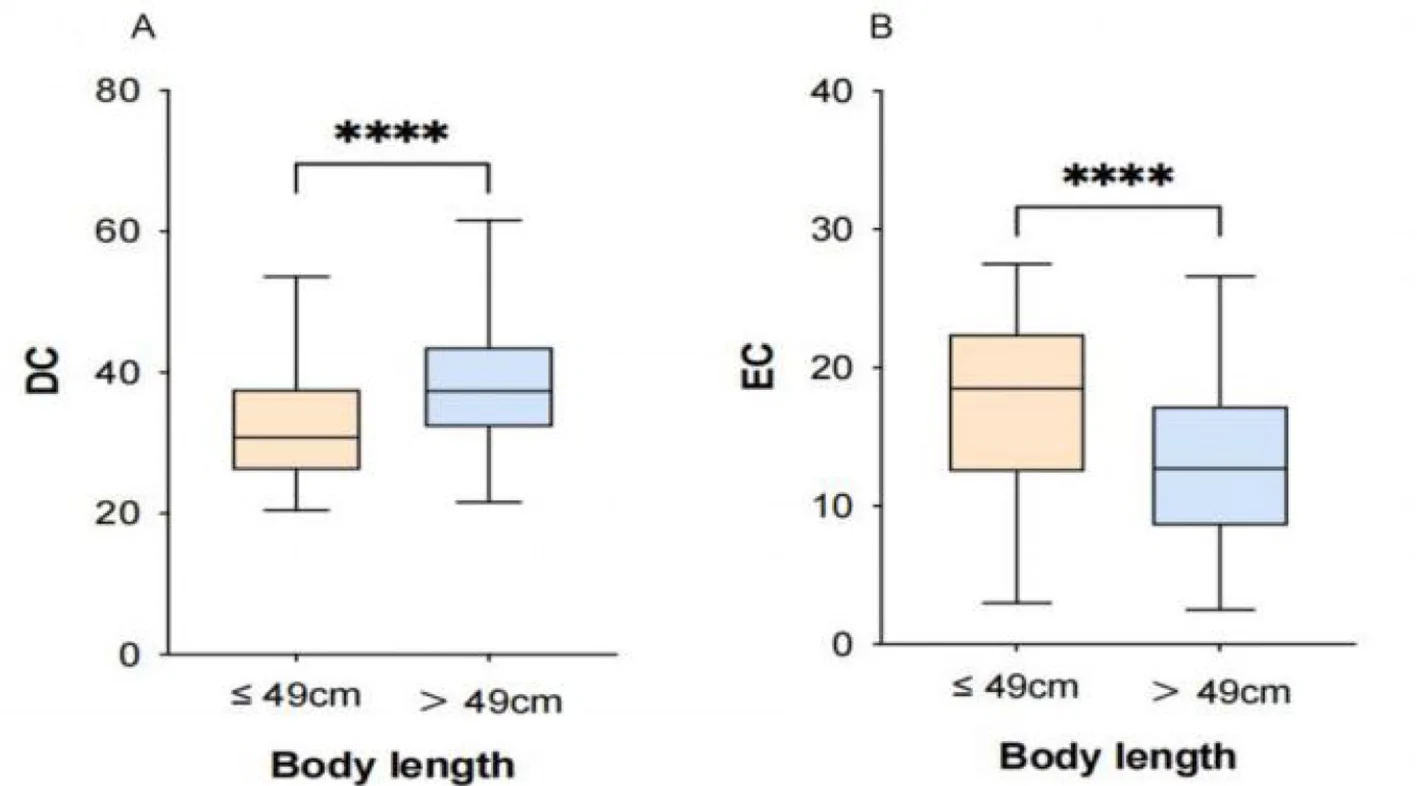
Differences in DC **(A)** and EC **(B)** among different body lengths. Statistical significance was assessed through Mann-Whitney test. DC, disturbance coefficient; EC, edema coefficient. ****means *P* < 0.001 after Bonferroni correction.

**Figure 8:**
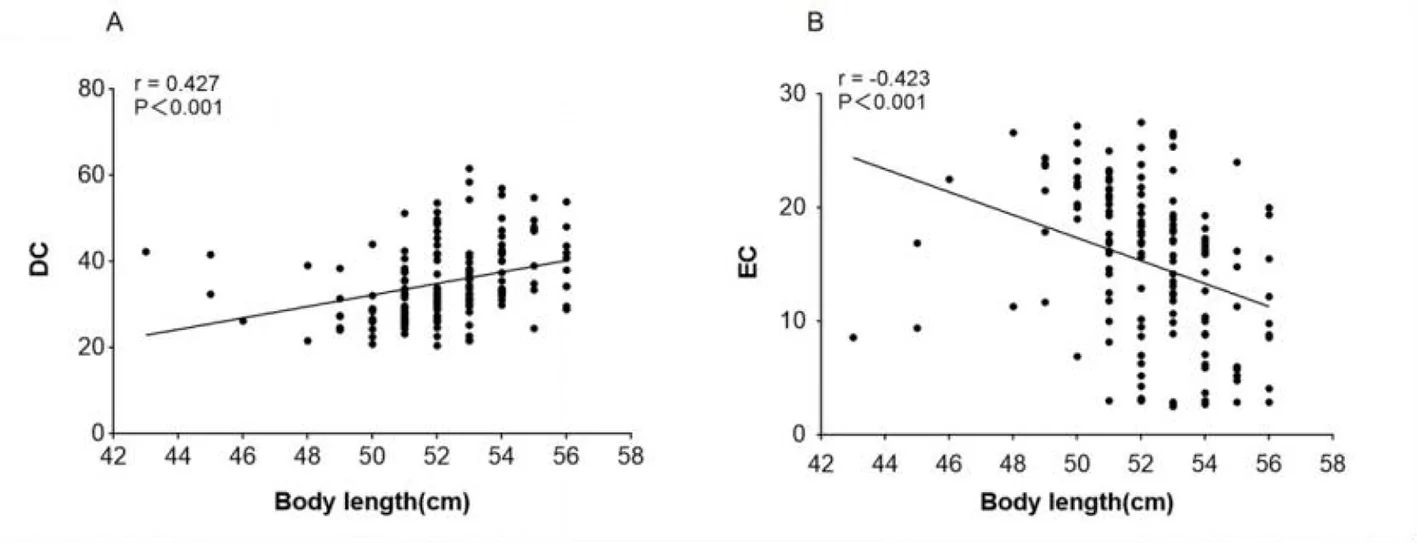
Association between body length and DC **(A)** and EC **(B)**. Statistical significance was assessed through Spearman's test. DC, disturbance coefficient; EC, edema coefficient.

### The correlation between DC, EC, sex, gestational age, and mode of delivery

As shown in Figure 9, no correlation was found between DC, sex, gestational age and mode of delivery. There was no association between EC, sex, gestational age and mode of delivery.

**Figure 9:**
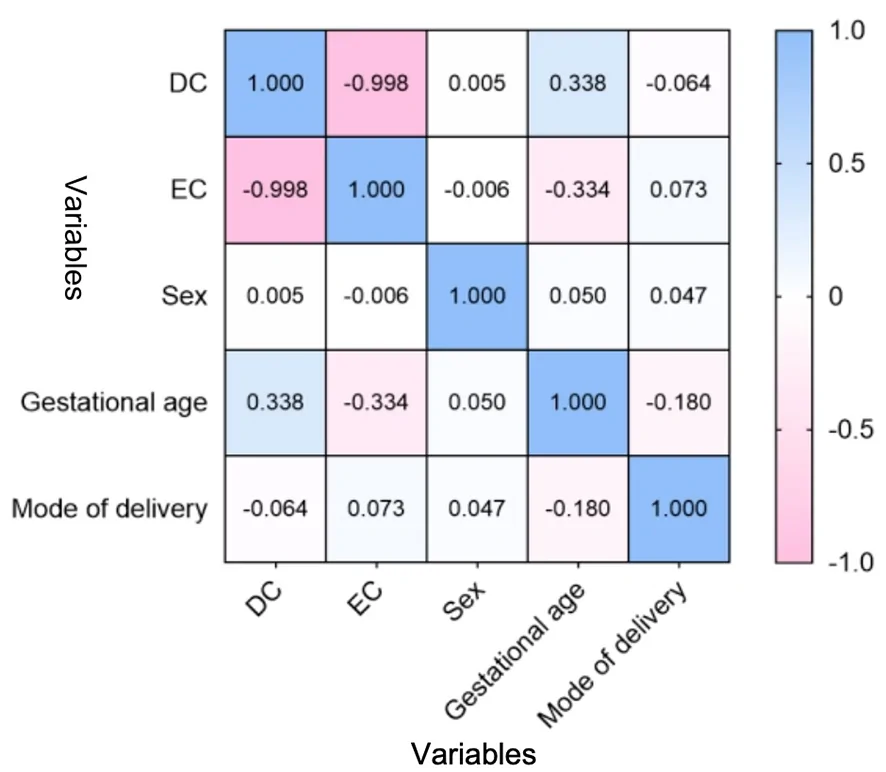
Correlation heatmap of DC, EC, sex, gestation age and mode of delivery. It reported Spearman's values, indicated by color scale ranging from −1 (pink) to +1 (blue). And obtained *P*-values were not statistically significant associations in both panels. DC, disturbance coefficient; EC, edema coefficient.

### Reference ranges for DC and EC in different groups

Based on the previous study, this study summarized the reference ranges for the included neonates, as shown in Table 2. We enrolled merely 7 neonates whose birth weight was over 4,000 g. We found the sample size failed to meet the requirement for establishing the 95% reference interval by the non-parametric percentile method; thus, we performed descriptive analysis for this subgroup using the median and interquartile range (IQR). For neonates with birth weight over 4,000 g, we observed that the median DC value was 47.40 (interquartile range, 40.80–58.50), and the median EC value was 4.80 (interquartile range, 2.70–9.80). Based on the previous study, this study summarized the interquartile ranges (IQR) for the included neonates, as shown in Table 3.

**Table 2:** Reference ranges for DC and EC in different groups.

Group	All No./total No.	95% reference interval of DC (*P*_2.5_-*P*_97.5_)	95% reference interval of EC(*P*_2.5_-*P*_97.5_)
Age, median (IQR), day			
0–1	150/150	21.60–55.84	2.86–26.60
1–2	150/150	22.94–62.91	2.50–25.85
1–3	150/150	23.53–67.88	2.20–24.75
Head circumference, median (IQR), cm			
29–32	50/150	24.13–49.20	5.93–23.0
32–34	50/150	27.40–55.03	3.93–19.10
34–36	50/150	29.07–59.40	3.0–16.03
Weight, median (IQR), g			
2,000–2,500	20/150	22.47–50.63	5.70–24.02
2,500–4,000	123/150	26.77–57.20	3.43–20.10
Body length, median (IQR), cm			
43–49	83/150	26.33–50.37	3.73–22.23
49–56	67/150	26.03–58.60	2.70–19.20

**Table 3:** Interquartile ranges (IQR) of DC and EC in different groups.

Group	All No./total No.	DC	EC
Age, median (IQR), day			
0–1	150/150	33.40 (28.45–41.60)	16.20 (9.88–20.38)
1–2	150/150	39.50 (32.57–46.20)	10.95 (5.57–16.90)
1–3	150/150	41.65 (35.30–46.90	10.13 (6.78–14.13)
Head circumference, median (IQR), cm			
29–32	50/150	33.73 (29.92–36.28)	16.42(14.55–20.63)
32–34	50/150	39.95 (35.82–44.58)	10.83(9.12–14.40)
34–36	50/150	44.37 (39.05–47.89)	8.87(6.65–12.08)
Weight, median (IQR), g			
2,000–2,500	20/150	27.70 (26.00–36.90)	21.15 (13.00–22.70)
2,500–4,000	123/150	33.50 (29.20–41.10)	16.10 (10.00–19.70)
4,000–4,350	7/150	47.40 (40.80–58.50)	4.80 (2.70–9.80)
Body length, median (IQR), cm			
43–49	83/150	30.80 (26.20–37.60)	18.50 (12.50–22.40)
49–56	67/150	37.40 (32.30–43.60)	12.70 (8.60–17.20)

## Discussion

Currently, research on non-invasive cerebral edema dynamic monitor is mainly focused on adults and older children, lacking sufficient evidence to monitor brain injury in neonatal setting. Our study aimed to bridge this gap (26, 27). First, we explored the correlation between head circumference, weight, and body length with DC and EC. Second, we established reference ranges for DC and EC in neonates of different ages, head circumferences, weights, and body lengths without traumatic brain injury (28). These results provide prospective evidence and empirical data for subsequent monitoring of neonatal brain injury.

When the intracranial contents of individuals across different ages change, they interfere with the propagation of electromagnetic waves. Alterations in the measured amplitude and phase of the waveform are manifested as variations in DC and EC, which can in turn reflect intracranial abnormalities (29). In adult studies, a DC value >155 indicated the possibility of intracranial hematoma or mass and was positively correlated with lesion volume. A DC value < 115 indicated the possibility of edema or water accumulation and was negatively correlated with its volume (30). In pediatric patients aged 6 years and older, DC ≤ 86.5 may indicate the presence of brain edema (16). A study involving traumatic brain injury-free children aged 0–16 years reported that DC increased with age in those younger than 5 years. Specifically, in children under 5 years old with a body weight of < 18.0 kg and a head circumference of < 51.0 cm, DC was positively correlated with age, weight, and head circumference, yet this study did not clearly specify such changes in neonates (18, 31). Another study on DC of normal children aged 1–16 years reached the same conclusion (23). In 1977, Reigel et al. (32) measured the brain impedance of 35 normal full-term neonates at birth and found it to be 32–36 Ω, which increased to 75–85 Ω by the age of 1 year. However, this study was conducted a long time ago, had a small sample size, and did not perform stratified monitoring. The main findings of our study on neonates with a gestational age of over 36 weeks are that DC and EC exhibited distinct patterns of change in the first few days after birth. Specifically, DC increased significantly from postnatal days 0–1 to days 1–2 and 2–3, while EC decreased over the same period. Our study also demonstrated correlations between DC and EC with head circumference, weight, and body length. DC showed a positive correlation with head circumference, weight, and body length, while EC exhibited a negative correlation with these parameters, similar to previous findings in traumatic brain injury-free children aged 0–16 years. These findings suggest that larger neonates (in terms of head circumference, weight, and body length) tend to have higher DC and lower EC. We derived DC and EC from the same raw bioelectrical impedance signals, which resulted in their strong negative correlation. We adopted different calculation formulas for these two parameters. Since they reflected distinct physiological characteristics of cerebral edema, they provided complementary clinical information. This may reflect potential physiological differences related to growth and development, as larger neonates may have more developed blood vessels and tissue structures, which could influence the distribution of edema and the degree of tissue disorder. Therefore, the importance of neonatal size and growth parameters should be considered when monitoring DC and EC.

Based on the research of the previous generation instrument in this experiment, sex had no significant effect on the disturbance index in healthy adults (18, 33). And studies in children had shown that DC was not related to sex (18, 19). This study is consistent with their research. In addition, we found no significant correlations between DC and EC with gestational age, and mode of delivery. Since this study included neonates of 36 weeks and above, neonates with smaller gestational age need to be further explored.

When interpreting our results, the limitations of the study should be taken into account. The neonates included in this study lived in the rooming-in unit with their mothers, and the short hospital stay of the mothers made it impossible to achieve a longer duration of monitoring. Given that this study was a single-center investigation, we inevitably encountered limitations imposed by factors such as climate and region, and we subsequently planned to increase the number of research centers and expand the sample size accordingly. We planned to conduct follow-up assessments of imaging findings and neurological development for all enrolled neonates in our subsequent research. Considering advanced maternal age, previous cesarean delivery history, pregnancy complications and maternal personal willingness for surgical delivery in recent years, we recruited a large number of neonates delivered by cesarean section in this study. We planned to increase the monitoring of vaginally delivered neonates in our follow-up research. In the subgroup analysis based on weight, some groups had small sample sizes, which may compromise the robustness of the results. Another limitation was that the study subjects were neonates over 36 weeks, and data on preterm infants were lacking.

## Conclusions

In neonates over 36 weeks, DC significantly increased from 0–1 day to 1–2 and 2–3 days after birth, while EC decreased. DC showed a positive correlation with head circumference, weight, and body length, while EC exhibited a negative correlation. DC and EC were not affected by sex, mode of delivery, and gestational age.

## Data Availability

The raw data supporting the conclusions of this article will be made available by the authors, without undue reservation.
